# The pediatric template of brain perfusion

**DOI:** 10.1038/sdata.2015.3

**Published:** 2015-02-03

**Authors:** Brian B Avants, Jeffrey T Duda, Emily Kilroy, Kate Krasileva, Kay Jann, Benjamin T Kandel, Nicholas J Tustison, Lirong Yan, Mayank Jog, Robert Smith, Yi Wang, Mirella Dapretto, Danny J J Wang

**Affiliations:** 1 Department of Radiology, University of Pennsylvania, Philadelphia, Pennsylvania 19104, USA; 2 Department of Neurology, University of California, Los Angeles, California 90095, USA; 3 Department of Radiology, University of Virginia, Charlottesville, Virginia 22904, USA

**Keywords:** Paediatrics, Network models, Brain imaging, Magnetic resonance imaging

## Abstract

Magnetic resonance imaging (MRI) captures the dynamics of brain development with multiple modalities that quantify both structure and function. These measurements may yield valuable insights into the neural patterns that mark healthy maturation or that identify early risk for psychiatric disorder. The Pediatric Template of Brain Perfusion (PTBP) is a free and public neuroimaging resource that will help accelerate the understanding of childhood brain development as seen through the lens of multiple modality neuroimaging and in relation to cognitive and environmental factors. The PTBP uses cross-sectional and longitudinal MRI to quantify cortex, white matter, resting state functional connectivity and brain perfusion, as measured by Arterial Spin Labeling (ASL), in 120 children 7–18 years of age. We describe the PTBP and show, as a demonstration of validity, that global summary measurements capture the trajectories that demarcate critical turning points in brain maturation. This novel resource will allow a more detailed understanding of the network-level, structural and functional landmarks that are obtained during normal adolescent brain development.

## Background & Summary

Neuroimaging has the potential to identify changes in the pediatric brain that may precede the development of neuropsychiatric disorders later in life^1^ and to quantify otherwise subjective symptoms such as pain^2^. Considerable effort has been devoted to building developmental brain templates across age^3–7^ in order to understand the dynamic processes associated with adolescence. However, relatively few datasets include consistent and cutting-edge structural and functional imaging modalities that are readily available and present few barriers to analysis.

Two major neurodevelopmental trends have been revealed by pediatric structural brain imaging. These include a continuous increase in the volume of white matter with age that has been attributed to myelination and improved connectivity between brain regions^8^ as well as an inverted U-shaped function for age-related variations in grey matter (GM), potentially the result of the ‘pruning’ of excitatory synaptic connections^9^ or intracortical myelination^10^. Giedd *et al.*^9^ reported prepubertal increase followed by postpubertal loss in GM volume in the frontal and parietal lobes, a general trend supported by subsequent studies^11,12^. Diffusion tensor imaging (DTI) has also been used to assess white matter integrity in the pediatric population^13,14^. Beyond normative data sets, brain morphometry analyses have also been successfully applied for probing neuroanatomical aberrations in various neurodevelopmental and neurogenetic disorders^1,15,16^.

Structural cortical and white matter trajectories provide only part of the picture in terms of neurodevelopmental processes that are important from childhood through adolescence. The function and metabolism of the pediatric brain has been relatively less explored than structural anatomy. To date, only a handful of nuclear medicine studies^17–20^ reported longitudinal developmental changes in cerebral blood flow (CBF). Current findings suggest a peak around 5–8 years followed by tapering to adult levels throughout adolescence. These patterns are, to a degree, consistent with structural MRI findings^13,21,22^. However, little research has been done to quantify the spatial and temporal relationships between function and structure as the brain develops.

The unique cross-sectional and longitudinal components of the Pediatric Template of Brain Perfusion (PTBP) will allow researchers to explore the relationships between brain structure, function and developmental landmarks, as well as environment. Calibrating normative relationships between structural and functional maturation is key to future work that may use neuroimaging to test for imminent psychiatric disorders or as an efficacy metric for emerging behavioral or pharmaceutical intervention strategies^23,24^. The PTBP is the first multi-modality functional/anatomical MRI template of the developing brain from prepubescence to adolescence based on longitudinal and cross-sectional data. The functional component of the template is based on quantitative cerebral blood flow (CBF) measurements using arterial spin labeling (ASL) perfusion magnetic resonance imaging (MRI)^25^ as well as blood oxygen level dependent (BOLD) neuroimaging data^26^. The structural component of the template includes both high-resolution T1-weighted neuroimaging, ideal for quantifying cortical structure^27^, as well as diffusion tensor imaging (DTI) for white matter quantification. DTI has been proposed as a valuable image-based biomarker in pediatric populations that may be used to stage tract myelination^28^. This confluence of modalities enables us to interpret neural development with respect to the cortical and white matter architecture that supports function and emergent cognition.

The host of imaging measurements provided by the PTBP is complemented by both socioeconomic and neuropsychological metrics. The novel PTBP dataset is publicly available and is accompanied by open-source processing tools^29^ that enable the measurements reported in this work to be reproduced by other scientists. In summary, this collection of data and standardized, open processing will both serve as a reference multiple modality dataset and shed light on the complex and multivariate process of adolesecent brain development.

## Methods

**Figure 1:**
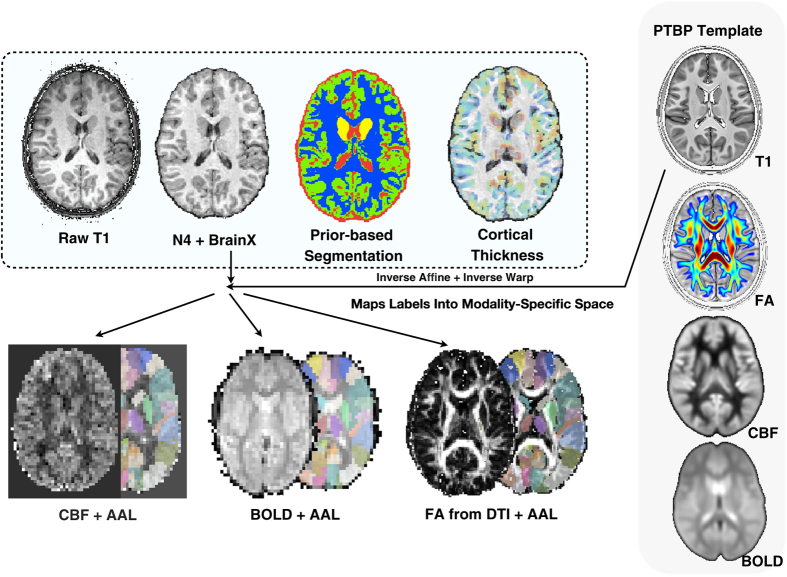
Overview of the PTBP. The multiple modality processing pipeline inputs and outputs. The population average T1 template is shown at upper right. The AAL labels are mapped to each modality and, along with 6-tissue segmentation, used to help compute summary measurements for all modalities and for multivariate prediction.

This document and all figures save Fig. 1 are generated on the fly by compiling the input file via the *R* and *LaTeX* interpreters using the *knitr* package. This produces the output pdf along with all statistical figures and quantitative demographic summaries.

### Cohort selection

The sample was recruited between January 2010 and February 2014. Overall cohort selection sought to match the demographic distribution of children ages of 7 to 18 years in the United States, based on US Census data, in terms of race, ethnicity, gender and family income. Data were collected at a single site, the Ahmanson-Lovelace Brain Mapping Center at UCLA, by the Laboratory of Functional MRI Technology (LOFT). Typically healthy developing children between 7 and 18 years of age were enrolled in this study. Exclusion criteria were designed to screen out children: (1) with previously diagnosed medical conditions (including developmental, neurological, psychiatric, and learning disorders); (2) who met criteria for a learning/psychiatric disorder or neurological condition not previously diagnosed; (3) who had been exposed to environmental conditions known, or likely, to impact normal development; (4) who cannot undergo an MRI scan; and 5) with limited English proficiency (as several tests to be administered as part of this study were not available in languages other than English). The complete list of exclusionary criteria is below:

- Developmental & Medical Disorders: Known developmental disorder (Failure to thrive; PKU etc.).
- Hearing impairment requiring hearing aid.
- Visual impairment (strabismus, visual handicap not correctable with regular glasses).
- Diabetes (Type I, treated with insulin).
- Systemic rheumatologic illness (for example, glomerulonephritis, endocarditis).
- Systemic malignancy requiring chemo- & CNS radiotherapy Congenital heart defect.
- Neurological Disorders, Seizure disorder.
- CNS infection (for example, meningitis).
- Brain tumor.
- History of closed head injury with loss of consciousness > 5 min.
- Muscular dystrophy or Myotonic dystrophy.
- Behavioral, Psychiatric, & Learning Disorders: Schizophrenia, Autism Spectrum Disorder, Bipolar Disorders, Recurrent Major Depression, Attention Deficit Hyperactivity Disorder (ADHD), Conduct Disorder, Tourette Disorder, Obsessive Complusive Disorder (OCD), Drug Dependence.
- Child Behavior Checklist (CBCL) subscale score <70.
- IQ rating <80.
- Achievement score >2 s.d. below age norms.
- Current/past language disorder (dyslexia, stuttering).
- Special education placement.
- MRI Contra-indications: Metal implants (braces, pins) or metal fragments, Pacemaker or electronic medical implants, claustrophobia, pregnancy.
- Limited English Proficiency.

Written informed consent to participate in the study and public release of the data was obtained from each subject and their parents according to the Institutional Review Board (IRB) of University of California Los Angeles (UCLA) guidelines prior to IQ assessment and MR scans.

### Assessment of behavior and environment

#### Psychometric evaluation

The Wechsler Abbreviated Scale of Intelligence (WASI)^30,31^ was administered to each subject resulting in three complementary measurements of IQ: the Verbal IQ (Vocabulary, Similiarities), the Performance IQ (Matrix Reasoning, Block Design) and the Full Scale IQ. Correlations between the full-scale IQ and verbal IQ is 0.89, between the full-scale IQ and performance IQ is 0.86 and between the performance IQ and verbal IQ is 0.54.

#### Assessment of socioeconomic status and parental background

**Figure 2:**
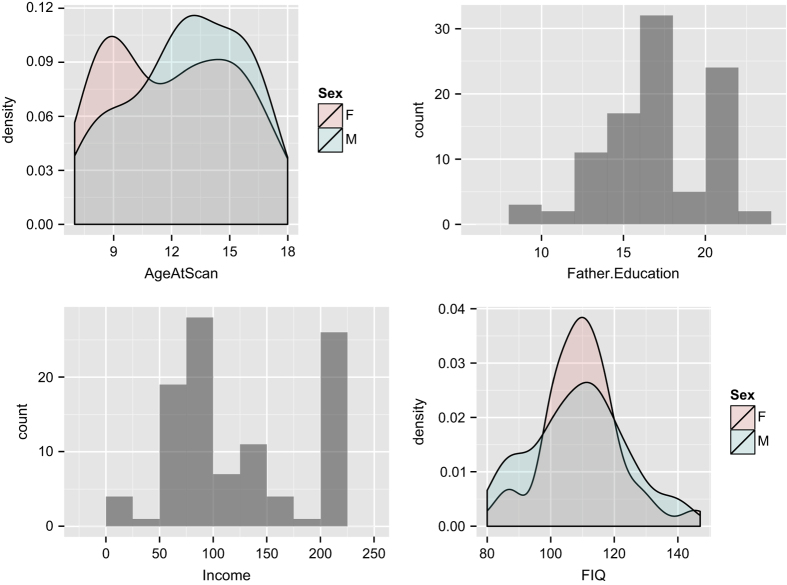
Overview of the PTBP demographics. The age distribution (12.4 +/− 3.12 years) for male (*n*=59) and female (*n*=61) subjects in the cohort is shown at top left. The histogram of paternal education (16 +/− 3.22 years) is at top right. The histogram of family income, in USD, across the full cohort is a bottom left. At bottom right is the histogram of full-scale IQ, grouped by gender, as measured by WASI (109 +/− 14.6).

Family income was quantified within a self-reported range defined in USD. The distribution of basic cohort demographics is shown in Fig. 2. Paternal and maternal educational levels were determined by years of education with, in this cohort, a minimum of eight years with several subjects having ≥20 years of education. These were subsequently translated into years of education for both parents, when available. Subjects also provided self-report Ladder scores on both the community and SES scales. The community scale identifies subject's perceived social standing within their local community. The Ladder-SES identifies subject's perceived wealth relative to society as a whole. Both sub-scales are in the range of 1 to 6 where values correspond to rungs on the ladder.

### Image acquisition methods

All MRI data were acquired on a Siemens 3 T TIM Trio scanner using the product 12 channel head coil.

#### T1-weighted acquisition

Magnetization-Prepared Rapid Acquisition Gradient Echo (MPRAGE) images were acquired using a 3D inversion recovery sequence with TR/TE/TI=2,170/4.33/1,100 ms. The resolution is 1×1×1 mm^3^ with a matrix size of 256×256×192. The flip angle=7° and total scan time was 8:08 min.

#### Cerebral blood flow

Pseudo continuous arterial spin labeled (pCASL) images were acquired using gradient-echo echo-planar imaging (EPI) with TR/TE=4,000/12 ms. The resolution is 3.125×3.125×6 mm (5 mm with 1 mm gap) over a 64×64×24 matrix. 40 label/control pairs were acquired. Generalized autocalibrating partially parallel acquisition (GRAPPA) was used with an acceleration factor of 2. Labeling duration was 1.5 s and the post-labeling delay was 1.2 s. Total imaging time was 5:30 min.

#### Diffusion tensor acquisition

Diffusion weighted images were acquired with single-shot multi-slice using spin-echo EPI with TR/TE=9,500/87 ms. A single b=0 volume was acquired along diffusion weighted images for 30 directions with b-value=1,000. The resolution is 2×2×2 mm with a matrix size of 128×128×75 voxels. The flip angle=90°.

#### BOLD acquisition

Blood oxygen level-dependent (BOLD) images were acquired in the resting state using 2D EPI with TR/TE=2,000/27 ms. The resolution is 4×4×4 mm over a 64×64×25 matrix and up to 244 time points were acquired. The flip angle=80°. Subjects were required to relax quietly while looking at a fixation point.

### Image processing methods

We employ Advanced Normalization Tools with *R* (ANTs*R*) http://stnava.github.io/ANTsR/ in order to check, organize and assemble a multiple modality database file that summarizes the range of measurements available in the PTBP. All scripts and source code are available in ANTs*R* and its dependencies.

ANTs*R* (answer) is an open-science analysis framework that seeks to enable predictive biomedical studies that integrate imaging modalities with other data. ANTs*R* resolves statistical needs with *R*, the statistical computing language, while using an ANTs core for image registration, segmentation and template construction^32–36^. This framework is uniquely general purpose in that it makes no assumptions about image content or organ systems to which it is applied and is appropriate for N-dimensional data (2-D, 3-D, 4-D). As evidence of this generality, ANTs*R* tools won two independent registration competitions, one in brain MRI^37^ and one in lung CT^38^. Furthermore, ANTs*R* is instrumental to one of the leading joint label fusion (multi-atlas segmentation) methods currently available for automated anatomical labeling^39^ and recently won open competitions in segmentation/prediction, BRATS 2013 at MICCAI 2013 (ref. 40) and the SATA challenges at MICCAI 2012 and MICCAI 2013, the latter of which employed several modalities across species and organ systems. At the same time, the framework is customizable such that it may be used to solve specific analysis problems by incorporating prior knowledge. We describe, below, how we employ ANTs*R* to: (1) provide a multi-channel population template representative of the modalities available in PTBP; (2) check data quality and validity of processing decisions in each modality; (3) compute summary measurements that enable biostatisticians and other researchers easy access to PTBP data with no burden of large-scale image processing. In the following section, we will report key summary measures that serve to support validity of both acquisition and post-processing. All software used to achieve these results is publicly available with significant efforts made to document both high-level use cases and individual tools. Releasing both data and post-processing streams, together, constitutes an open-science approach increasingly recognized as critical to the advancement of science^41–43^.

#### Multiple modality population template

Normalizing images to a standard coordinate system reduces intersubject variability in population studies, allows coordinates to be compared across studies and allows one to employ prior-based segmentation/labeling techniques. The latter are important for tissue segmentation, brain extraction, cortical parcellation and functional or structural connectivity studies. Various approaches exist for determining the normalized space such as the selection of a pre-existing template based on a single subject, for example, the Talairach atlas^44^, or a publicly available averaged group of subjects, for example, the MNI^45^ or ICBM^46^ templates. We employ the symmetric groupwise normalization method (SyGN) of ref. 47 which explicitly models the geometric component of the normalized space during optimization to produce brain images that represent the population under study in terms of both the shape of anatomy and the appearance of anatomy. Coupling the intrinsic symmetry of SyN pairwise registration^33^ and SyGN's optimized shape-based averaging of the template appearance leads to a powerful framework for population-specific imaging studies across modality and species^47–51^. We achieve this generality of application by storing population-specific prior information within the template space to aid with brain extraction or other quantification steps.

Traditionally, this approach was used for single modalities but, more recently, is extended for multiple modalities via cohort-specific templates that capture the average shape and appearance of T1, DTI and functional images, as in refs 25, 32, 49, 52. Some of these templates have been released for public use^29^ and include population averages with variation across both age and modality. While these resources are of value, we follow the philosophy of building a population-specific template for the PTBP and its modalities such that we take advantage of the latest image registration methodology.

Our template building procedure first constructs an average T1 brain image from the full population (*n*=119). We then extract the template brain based on multi-template labeling^39,53^, currently the state-of-the-art for automated labeling. Using a similar procedure, we generate probabilistic tissue/structure priors for each of the 6 tissues of interest: cortex, deep gray matter, cerebrospinal fluid, white matter and the cerebellum. A summary of the template building procedure is available, with two example datasets, at https://github.com/ntustison/TemplateBuildingExample (this is a pedagogical usage example, not an archived dataset or published software). The same methods constructed the templates available at ref. 29 which we used as a starting point for PTBP custom tissue prior generation. Once the average image and priors are completed (file PTBP.zip), one may transfer all of the data by a small deformation registration into the standard MNI space^54^ (file PTBP_MNI.zip).

#### Template-based processing of individual subject T1 images

An overview of the ANTs-based structural pipeline is available in ref. 29. Given a finalized T1 template, we are able to segment and process the individual T1 data. We subsequently process individual modalities including DTI, ASL and BOLD. Finally, we generate the geometric transformations from the companion modalities to the core T1 anatomical image as well as the template space. We achieve this after the full multiple modality dataset is processed, as described below. In total, the steps involved for processing an individual multiple modality dataset are detailed here http://jeffduda.github.io/NeuroBattery/, along with an example dataset and template that may be used to verify reproducibility of the procedure. This is repeated for each individual in a population. We now provide an overview of the processing steps for T1 and then for each modality. Figure 1 summarizes the full pipeline.

#### T1 brain extraction

Brain extraction using ANTs combines template priors, high-performance brain image registration^33^, and Atropos^32^ with topological refinements based on mathematical morphology, as described in ref. 29 and implemented in antsBrainExtraction.sh. An example use case is here https://github.com/ntustison/antsBrainExtractionExample (this is a pedagogical usage example, not an archived dataset or published software). Briefly, In this stage, we use a coarse and relatively fast registration to calculate a warp between the template whole-head image and the subject of interest. This enables us to warp a probabilistic brain extraction from the template into the subject space. The warped template probability map is thresholded at 0.5 and the resulting mask is dilated with a radius of 2. Atropos is then used to generate an initial 3-tissue segmentation estimate within the mask region. Each of the three tissue masks undergo specific morphological operations which are then combined to create a brain extraction mask for use in the rest of the cortical thickness workflow. A comparison using open access brain data with publicly available brain extraction algorithms including AFNI's 3dIntracranial^55^, FSL's BET2 (ref. 56), Freesurfer's mri_watershed^57^, and BrainSuite^58^ demonstrated that our combined registration/segmentation approach^47^ performs at the top level alongside BrainSuite (tuned) and FreeSurfer.

#### Six tissue segmentation

Our segmentation procedure leverages two methods that are guided by anatomical priors and that work in concert to parcellate the brain into six anatomical/tissue classes: cerebrospinal fluid, cortical gray matter, deep gray matter, white matter, brain stem and cerebellum. These tissue priors are first mapped into the subject space by performing a large deformation SyN registration between the template cerebrum and the individual subject cerebrum, as in refs 37, 42, 59. The deformed template priors are then used to guide expectation-maximization (EM) segmentation (Atropos prior-based segmentation^32^) alternated with inhomogeneity correction via the N4 bias field correction algorithm^34^. The name N4 derives from the fact that it is an improvement on the classic N3 algorithm^60^. Atropos refers to a figure in greek mythology who wielded ‘shears of fate’; in the case of segmentation, the shears refer to dividing the image into parts. Due to the important interplay between segmentation and bias correction, we perform multiple N4 ⇔ Atropos iterations. In order to better integrate Atropos and N4, we use a pure tissue probability weight mask generated from the posterior probabilities generated from the segmentation process. This procedure is described in more detail in refs 29 and 32. A reproducible example segmentation dataset and command line for the ANTs method antsAtroposN4.sh is here https://github.com/ntustison/antsAtroposN4Example (this is a pedagogical usage example, not an archived dataset or published software).

#### DiReCT (aka KellySlater/KellyKapowski) cortical thickness estimation

Brain segmentation enables us to compute cortical thickness via the Diffeomorphic Registration-based Cortical Thickness (DiReCT) algorithm. DiReCT was introduced in ref. 61 and made available in ANTs with the program KellySlater. Since then several improvements have been made and incorporated into the program KellyKapowski, in particular determining an automated parameter set based on extensive experimentation and comparison with reference cortical thickness values available in the literature. Importantly, we recently showed that these methods outperform the FreeSurfer package^62^ in terms of extracting information from cortical thickness that is predictive of gender and age in a large collection of neuroimages across the lifespan^29^. Among the most significant advancements is that the more recent implementation is multi-threaded, written in rigorous ITK coding style, and has been made publicly available through ANTs complete with a unique user interface design developed specifically for ANTs tools. An example use case with reference two-dimensional data is in https://github.com/ntustison/antsCorticalThicknessExample (this is a pedagogical usage example, not an archived dataset or published software). Ultimately, the DiReCT algorithm produces an image, in subject T1 space, that contains voxel-wise measurements of cortical thickness that are appropriate for use in voxel-based or region-based statistical studies as in refs 50, 63–65.

#### Regional brain labeling with AAL labels

The final step in our T1 pipeline involves producing summary measurements for overall brain volume, the six tissue segmentation and for regional and lobar cortical thickness. We employ the widely used AAL labelset for this purpose^66^. These labels are transformed from our high-resolution group template space into the individual subject space by following the inverse of the template diffeomorphic mapping. AAL contains standard regional parcellations of the cortex, cerebellum and deep gray matter structures including the putamen, thalamus, hippocampi and caudate. For each subject that we process, we measure these volumes and regional average thickness producing a single row of information which is ultimately entered into a summary demographics file. This is used not only for performing studies of cohort variables but also for initial data checking that is, identifying subjects with global or regional outlier values with respect to known reference values.

#### DTI processing

Our procedure for analyzing DTI with ANTs was validated in ref. 67. In brief, we have developed an automated processing pipeline for diffusion imaging using the open source tools Camino^68^ and ANTs, which provides pre-processing, brain extraction, diffusion tensor computation, and normalization to template space, as well as diagnostic images in the subject space to aid quality control, including fractional anisotropy, average corrected DWI, and noise variance. The core of the method is to, first, process data in DWI space and, subsequently, transform the data into individual T1 or group template space.

The first step in DTI processing is to perform motion and distortion correction of the diffusion weighted images. The first unweighted image in the diffusion sequence is used as the reference image for motion and distortion correction. The remaining unweighted images are rigidly aligned to the reference image and averaged; this average image is used as the reference image for affine correction of the diffusion-weighted images (DWI) for motion and distortion caused by the diffusion weighting gradients. A brain mask is computed by aligning the average DWI to a template, and warping the template brain mask into the subject space. Processing then continues on the brain-extracted image. Diffusion tensors are calculated using an iterative weighted linear least squares algorithm^69^.

The transformed DTI are warped to the template space by combining the intra-subject DWI to T1 warp with the warp previously defined to normalize the subject's T1 image to the template space. The correct anatomical orientation of the diffusion tensors is preserved by applying the preservation of principal direction method^70^, and scalar statistics such as FA and mean diffusivity are computed from the normalized diffusion tensors.

#### Estimating nuisance variables in functional images

Several steps are common to processing either BOLD or ASL functional MRI. These include brain extraction, motion correction and nuisance variable estimation. Over the last one to two years, we introduced methods into ANTs for estimating these parameters from 4D time series data. Motion correction is performed by the antsMotionCorr program in ANTs which uses a mutual information similarity metric and a nonlinear conjugate gradient optimizer to maximize the Affine or Rigid similarity between each image in a time series and a sequence specific reference image. These methods are based on the Insight ToolKit version 4 revision, as described in ref. 71. The motion parameters for each time frame are written out to a CSV file such that they may be summarized and possibly used as nuisance variables within population-level statistics^72^. We make different choices of reference image for each modality. For BOLD, we choose the average image from the full time series. For CASL and pCASL, we choose the average control labeled image. For PASL, we use the acquired M0 image. A brain mask is computed by either a simple morphology procedure (morphological erosion followed by largest component selection and morphological dilation) or by aligning the reference motion correction image to a sequence-specific template and warping the template brain mask into the subject space. For the PTBP, we used the latter approach.

The second major nuisance, beyond motion, is physiological noise^73–75^. While one should select optimal nuisance parameters for each study and based on data quality, we elect to use the data-driven and automatic CompCor approach^75^ which was validated in ASL and BOLD and does not rely on anatomical segmentation. The approach performs singular value decomposition on high temporal variance voxels and uses (typically 3 to 6) singular vectors as nuisance regressors. These regressors were shown to capture physiological motion, scanner noise and other factors that are unrelated to cerebral blood flow or BOLD activation. Both motion correction and CompCor estimation with ANTs are shown here https://github.com/stnava/fMRIANTs (this is a pedagogical usage example, not an archived dataset or published software).

#### Cerebral blood flow (CBF) from ASL

Parenchymal perfusion is an important physiologic parameter in the evaluation and management of brain disorders as well as a surrogate index of neural activity^76^. ASL perfusion MRI is also ideally suited for pharmaceutical trials in pediatric populations as it allows absolute CBF quantification, is totally noninvasive and is potentially sensitive to treatment response^23^. In contrast, neuroanatomically defined measurements such as cortical thickness^22,77,78^ and FA derive directly from a relevant physical property of the imaged brain tissue and may fail to capture shorter term functional effects due to intervention or training. The PTBP establishes the feasibility of ASL-MRI in an age range during which the brain is rapidly developing and when early signs of future neuropsychiatric disorders may emerge. This functional quantitative measure (versus the relative values provided by BOLD) has the potential to reveal alterations in the brain due to injury^79^, pain^80^, pharmacological intervention^81,82^ or that precede visible structural change and may indicate cortical reorganization^83^. CBF is a more repeatable functional measurement than BOLD^84,85^, may be used in network analysis in lieu of or combination with BOLD^86^ and provides a unique view on the brain complementary to DTI and T1.

Each subject's M0 image, obtained as the mean of the control images, was warped to the subject's T1 image using the antsIntermodalityIntrasubject.sh script. These transforms were concatenated with the subject-to-template transforms to warp the template labels to the subject native ASL space. The M0 image served as a reference for motion-correction of all time-point volumes. In addition to the motion and nuisance regressors described above, we included either the tag or control label of the image as a regressor, with the coefficient of that regressor corresponding to the average difference between tag and control. All regressors were included in a robust regression scheme for CBF calculation^87^. The equation for CBF calculation can be found in ref. 88 with an assumed labeling efficiency of 0.85. Full details are available in the open-source script at https://raw.github.com/stnava/ANTs/master/Scripts/antsASLProcessing.sh (this is available as part of the Figshare repository, Data Citation 1). The blood T1 value was adjusted for age and gender as T1=(2115.6–21.5*age-73.3*sex) ms, where female sex was set to 0 and male was set to 1, as suggested in ref. 88.

#### Network analysis with resting BOLD fMRI

Network analysis may be performed with any of three types of input time series data: standard BOLD, ASL-BOLD or ASL-CBF. The ASL-CBF signal may have advantages over BOLD particularly in the orbitofronal and anterior temporal regions where standard BOLD signal dropout occurs^89,90^. However, as ASL-CBF network analysis is relatively new and we have other methods of validating ASL-CBF, we focus on network analysis using the resting BOLD modality from the PTBP. The goal is to extract standard network measurements that may be assessed for comparison with demographics to help establish validity of the acquisition and processing.

We base our graph/network construction on the standard AAL cortical labels which we transform from the T1 subject space to the BOLD space. We prefer to perform analysis in the BOLD space to minimize the confounds associated with interpolation and resampling of low resolution imagery as these may bias the results in a subject-dependent manner. Each BOLD image is first residualized with respect to the baseline nuisance parameters described above, that is, motion parameters and CompCor singular vectors. For each AAL label of interest (here only the cortex, labels 1–90), a region-averaged time-signal is calculated. Each time-series is then bandpass filtered using the Christiano-Fitzgerald filtering^91^, as implemented by the *R* function cffilter in package *mFilter*, to examine a range of frequencies appropriate for the specific data type. For band-pass filtering, we select the frequency range 0.01–0.1 Hz based on prior work^92^ and on preliminary analyses, in a few subjects, of network reproducibility. The network component of the processing pipeline is available in the ANTs/ANTsR script antsBOLDNetworkAnalysis.R which assumes that brain labels and motion parameters are available. The primary output of this script used in data checking, below, is the N×N Pearson correlation matrix defined by the correlation between the filtered time-signals for each of the N labeled regions.

## Data Records

Within the neuroimaging community, it is advantageous to release both reconstructed volumetric or spatiotemporal images in a commonly used processing format (often the NIFTI format) along with some post-processing and summary measurement data to help serve different reuse cases and communities that do not have the ability to process images at a large scale. We therefore present PTBP data as both easy to analyze summary measurements and raw NIFTI images at *figshare* (Data Citation 1). An overview file summarizing subjects and modalities is in ‘ptbp_data_index.csv’. The raw imaging data is organized by subject identifier (for example, PEDSXYZ) where PEDS is constant and X, Y and Z are each numerals in 0–9. Within each subject folder is multiple modality imaging data organized by date of acquisition and, at the last level, by imaging modality. Each medical image is shared within the NIFTI data format, a common anonymized data format ready for image processing. All released data is in raw nifti form. An example subject folder is:

ls PEDS012/*/*

PEDS012/20121031/Anatomy:

PEDS012_20121031_mprage_t1.nii.gz

PEDS012/20121031/BOLD:

PEDS012_20121031_bold_fc_1.nii.gz

PEDS012/20121031/CBF:

PEDS012_20121031_meanbold.nii.gz

PEDS012_20121031_meancbf.nii.gz

PEDS012/20121031/DWI:

PEDS012_20121031_0013_DTI_1_1x0_30x1000.bval

PEDS012_20121031_0013_DTI_1_1x0_30x1000.bvec

PEDS012_20121031_0013_DTI_1_1x0_30x1000.nii.gz

PEDS012_20121031_0019_DTI_1_1x0_30x1000.bval

PEDS012_20121031_0019_DTI_1_1x0_30x1000.bvec

PEDS012_20121031_0019_DTI_1_1x0_30x1000.nii.gz

PEDS012/20121031/PCASL:

PEDS012_20121031_pcasl_1.nii.gz

These modalities include the T1-weighted MRI mprage_t1, the PCASL time series image pcasl, its derived CBF image meancbf and the BOLD time series image bold_fc. The DTI dt is accompanied by derived images including the RBG showing principal direction rgb, the fractional anisotropy fa, the mean diffusion md, the average diffusion weighted image (DWI) dwi and the estimated (approximate) DTI-space brain mask brainmask.

These data are quantified within a demographics file named ptbp_summary_demographics.csv that is indexed by the same subject identifiers. This primary data includes cortical thickness based on T1 segmentation, BOLD connectivity, fractional anisotropy DTI measurements and cerebral blood flow. A population summary is in Fig. 2. We also summarize the raw neuroimaging data values across all subjects for structural modalities in Supplementary Figs 1 and 2 and functional modalities in Supplementary Figs 3 and 4. We quantify the T1-weighted imaging using regional cortical thickness values derived from AAL. The column names of this label set are of the form ThickMeanAALLabelName. The range of thickness values is largely within the expected range that is, between 1 and 5 mm^93^ when using 1 mm^3^ neuroimaging. In similar manner, we quantify the DTI using fractional anisotropy within the six tissues of our brain segmentation. The column names of this label set are FAMeanTissueLabelName and show age-related increase, as expected^94^. We quantify the ASL-derived cerebral blood flow measures using regional AAL labels. The column names of this label set are CBFMeanAALLabelName. The range of CBF values is largely within the expected range that is, between 50 and 120 ml per 100 grams per minute similar to values reported previously in ref. 25. Reduction in CBF occurs both globally with age and specifically in the inferior temporal cortex, precuneus, middle frontal gyrus and insula. We also quantify the BOLD connectivity by the mean correlation of each AAL node with all other nodes described in the methods section. The column names of these mean BOLD correlation levels are BOLDAALLabelName.

### Imaging data collection summary

We made every effort to collect data that is complete in terms of both demographic measurements and imaging measurements for all *n*=120 subjects. However, some subject data could not be obtained or did not reach adequate quality. One subject is missing 8 timepoints of T1 data; There are 25 missing DTI datasets, 30 missing CBF datasets and 43 missing BOLD datasets. Of *n*=183 total datasets acquired, *n*=118 had complete datasets which is defined as having T1, DTI, CBF and BOLD data at baseline. 6, 13, 9 and 37 subjects had T1 data with 4, 3, 2 and one image collected, respectively. Note that to facilitate analysis of the largest possible dataset, one may employ the ANTs*R* function antsrimpute in order to impute missing data. However, our analyses below do not use imputed imaging data. Furthermore, note that our released summary demographics file details, for each subject and time point, which modalities are present or absent.

## Technical Validation

We check for expected statistical outcomes to establish the biological plausibility of PTBP behavioral and neuroimaging measurements. These analyses focus on global metrics of validity and reveal cross-sectional trends that are largely extant in the literature. To achieve this, we employ *R*, a popular programming/scripting language that is designed to make advanced statistical analysis accessible and support free and reproducible statistical studies. When combined with the image processing utilities available in ANTs, *R* provides a convenient and powerful interface for performing common statistical analyses of imaging data. In addition, *R*'s standardized syntax minimizes the learning curve for performing a wide variety of different analyses. The basic form of a statistical model in *R* is

(1)Outcome≈Predictor1+Predictor2+Predictor3+…

Factor and continuous variable predictors can be combined seamlessly, and a wide variety of model types, including linear models with Gaussian noise, logistic, and Poisson models are available. We use such models with *R*'s lm function to assess both imaging and demographic models. When performing region-of-interest (ROI)-based analysis of the relationship between imaging data and cognition, one typically averages the voxel values within a given ROI and then tests the averaged values against a predictor. In *R* syntax, this is written as

(2)ROIvalue≈cognition+nuisancedemographicvariables.

We follow a similar strategy to technical validation of our baseline demographic measurements and testing for known relationships between ROI values derived from neuroimaging, age and gender.

### Basic demographic relationships

The PTBP demographics include age, gender and handedness. The PTBP also includes self-reported measures relating to environmental experience such as family income, Ladder SES scores, WASI IQ scores and parental educational level. Here, we detail basic relationships between these variables as evidence of appropriate data curation.

The relationship between parental education and IQ metrics during adolescence is well-established^95^. The PTBP recapitulates this relationship, as expected, when using a model of the form IQ ≈ age+gender+LadderSES+P.Edu+rank(Income), where ‘rank’ indicates a rank transform. The *P*-value for the relationship between paternal education and performance IQ is 0.0012426. The *P*-value for the relationship between paternal education and verbal IQ is 0.001403. Furthermore, the Ladder SES score relates to family income, as expected, with *P*-value 0.0254036. The regression equation for the LadderSES and income relationship is (using *R* syntax) LadderSES ≈ age+gender+FIQ+P.Edu+rank(Income).

### Multiple modality neuroimaging quality assurance

We employ procedures described in ANTs documentation https://github.com/stnava/ANTsDoc for rapid visual inspection of image registration and segmentation results.

### Multiple modality repeatability

We previously verified the repeatability of our structural measurements in ref. 29, the specificity and sensitivity of our DTI analysis protocol in ref. 67, reliability of BOLD network measurements in ref. 92 and ASL-CBF in ref. 25. In the latter work, we verified that CBF repeatability achieved intraclass correlation (ICC) of 0.65. ICC of cortical thickness based on structural segmentations is 0.98 (ref. 29).

### Multiple modality neuroimaging predictors of age

**Figure 3:**
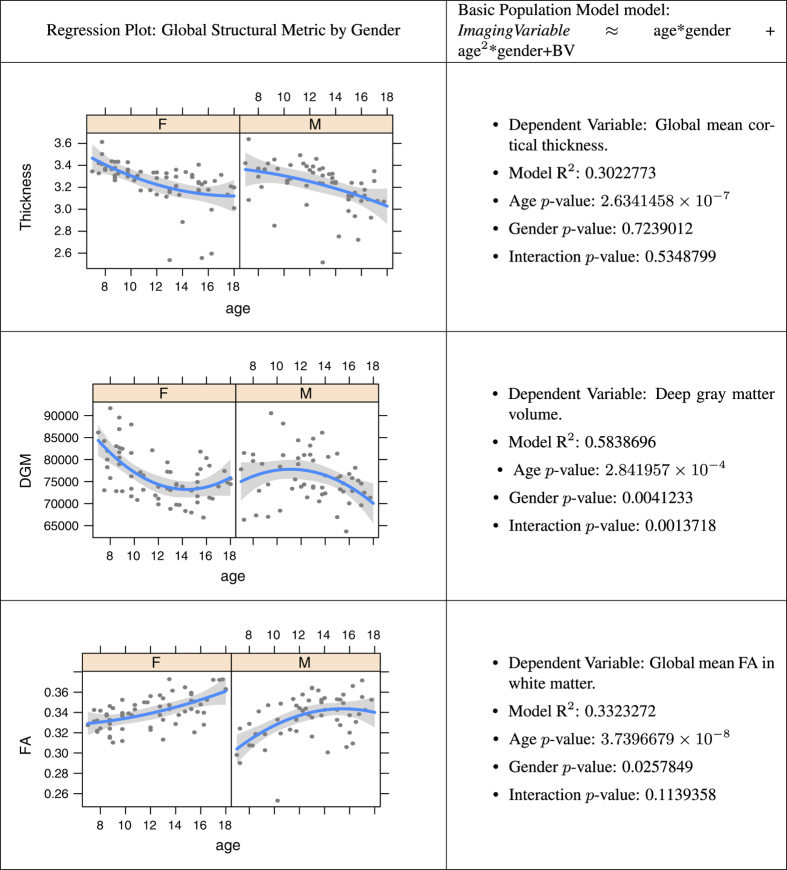
Age modeling from structural imaging variables. Relationship of global structural measurements, age and gender for each modality while controlling for brain volume (BV, above). *n*=91.

**Figure 4:**
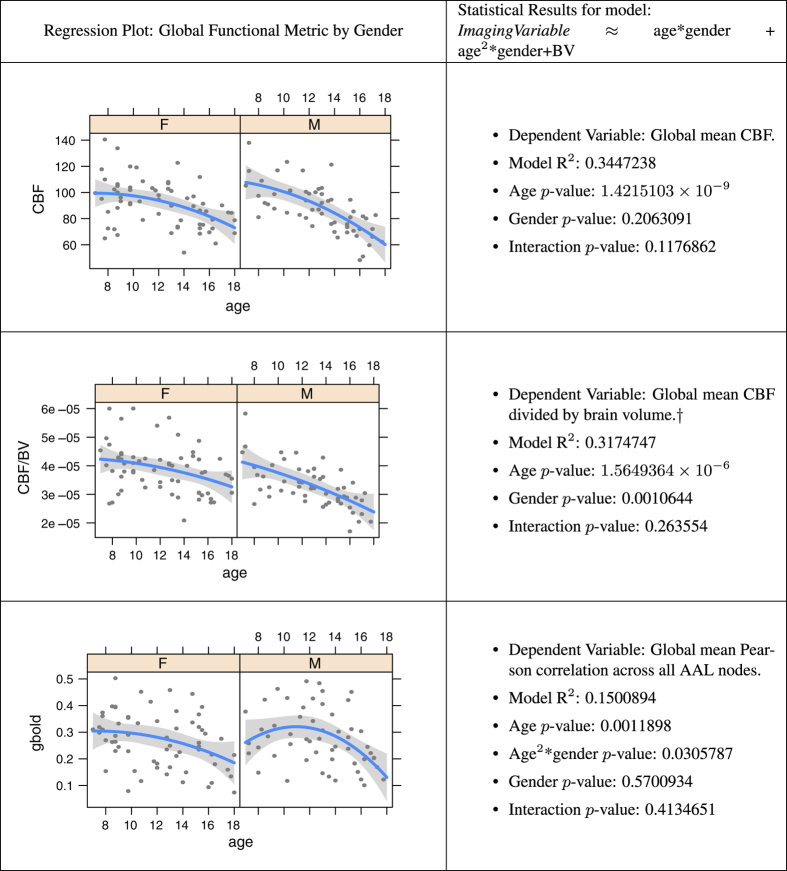
Age modeling from functional imaging variables. Relationship of global functional measurements, age and gender for each modality, controlling for brain volume within the regression, except where noted by a dagger †. *N*=91.

**Figure 5:**
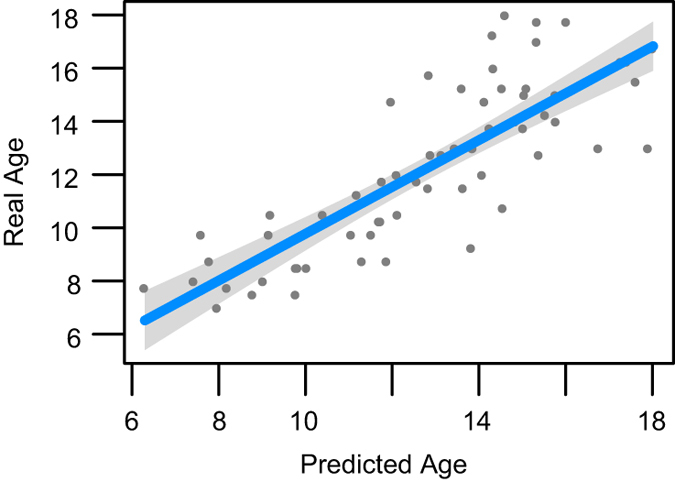
Age prediction from all imaging variables. We train on half of the data and predict age in the other half of the data using a general linear model age ≈ BStem+CBF+FA+Thickness+DGM. See Table 1 for evidence that each of these predictors is important to the model. We obtained this predictor set by performing a supervised backward variable elimination in the regression on the training data. *n*=91 for the full data-set, split into testing and training sets of 45/46 100 times, to validate predictive relationship of multiple modalities with age.

The insights gained from multiple modality MRI inform our understanding of brain network elaboration during childhood and adolescence. Observed neuroanatomical changes in the brain during maturation may be attributed to many different mechanisms^96,97^, such as: increasing myelination, neuron production, decreasing cortical thickness due to pruning of neural cell bodies, changes in axonal caliber, pruning of fiber tracts and axonal branching. Developmental trajectories of these processes are likely influenced by both endogenous and exogenous variables. Here, we establish that global summaries of each PTBP imaging modality relate to basic descriptors of the cohort. Figures 3 and 4 summarize relationships between global structural and functional imaging metrics, age and gender. Table 1 and Fig. 5 show how these global summary imaging variables, acting within the same model, predict age in a training-testing paradigm. This latter analysis is similar to ref. 98 which assesses age relationships across several modalities in a large multiple modality study and results in similar accuracy to that reported here. In the studies below, we use *n*=91 of *n*=120 subjects that have complete, reasonable quality multiple modality datasets: T1, DTI, BOLD and ASL-based CBF.

### Multiple modality neuroimaging predictors of IQ and Ladder-SES

Following on the model built, above, for age, we report that regression results of the form Full-scale IQ ≈ BrainStem+Thickness+FA+DGM+CBF reveal that cortical thickness is a significant predictor of FIQ (*P*<0.004). Similarly, we report that regression results of the form Ladder-SES ≈ BrainStem+Thickness+FA+DGM+CBF reveal that deep gray matter is a significant predictor (*P*<0.0007). These results suggest the validity of PTBP data collection strategies.

### Cortical thickness, age and gender

In recent years, a number of groupwise structural morphometry studies have used cortical thickness to study neurodevelopment and other causes of cortical plasticity^5,9,11,99,100^. Cortical thickness quantifies the width of the cortical sheet from its boundary with the white matter to its nearest boundary with the cerebrospinal fluid. Image-based thickness, typically reported in millimeters, links directly to findings in histology and post-mortem data, as well as existing normative values available in the literature^9,11,12,22^. We analyzed the global mean cortical thickness with respect to age and gender. The PTBP data confirms age effects in cortical thickness, though does not suggest an interaction. Overall, the rate of global cortical thinning across genders is similar. Similar effects have been found with more localized analysis^11,12,101,102^. Note, however, our data validation model explicitly controls for brain volume within the regression.

### Deep gray matter volume, age and gender

We define deep gray matter as in ref. 29 where it includes caudate, putamen and the thalamus. The deep gray matter relationship with age is significantly different between males and females. Males show an inverted U curve with age that peaks near age 11 whereas females exhibit a U-shaped curve with a minimum near age 14. There is little background research on this topic and we note, simply, that it perhaps deserves additional investigation in future work. As cross validation, we tested the same model in a separate dataset provided by the Nathan Kline Institute and found a similar trend.

### White matter integrity, age and gender

Eluvathingal used the FA to investigate sex differences in white matter development and found no significant differences between males and females in the age range of 6–17 (ref. 103). The PTBP suggests that FA increases rapidly with age in both genders and is slightly higher in females. Furthermore, as seen in Table 1, FA contributes different information to age prediction than cortical thickness, as suggested in ref. 102.

### Cerebral blood flow, age and gender

The mean PTBP CBF in cortex is 96.5, in white matter is 32.7 and in deep gray matter is 86.1. Normative perfusion data in both control and pathological states has been sparse in the pediatric population, in part because traditional perfusion measurement has relied on radioisotopes and contrast agents. Primarily, raw cerebral blood flow relates strongly to age with inflection points that appear to be coupled closely with puberty^104,105^. In the PTBP, the inflection points for cerebral blood flow appear to occur before age 8. Even in the presence of several other modalities, cerebral blood flow is strongly predictive of age. Gender effects do not appear to be significant in raw CBF though do emerge when dividing the global CBF by brain volume. However, further elaboration of these measurements might be gained by the use of partial volume correction or voxel-wise or regional measurements. There is little impact of the brain volume nuisance variable on the relationship of CBF with age.

### BOLD connectivity, age and gender

BOLD-based network connectivity analysis is a recent development^106^ and remains controversial^107^ due to the many confounds in the acquisition and subsequent analysis^75^, in particular motion^72^. However, several groups successfully use BOLD to establish the consistent presence of the default mode^108^ and salience networks^109^ and to quantify relative levels of connectivity across brain regions^110^. We investigate global levels of BOLD correlation with the PTBP and find evidence that is consistent with the limited extant analyses of adolescent network development^111^ that is, age-related connectivity changes, gender differences between males and females in connectivity levels and, finally, greater connectivity in default mode regions. However, the global Pearson correlations used here have limited interpretability and may be confounded with motion or other variables despite our efforts to control for these effects via standard volume censoring^72,112^.

## Usage Notes

We recommend data processing with Advanced Normalization Tools and *R* (ANTs http://stnava.github.io/ANTs/ and ANTs*R* http://stnava.github.io/ANTsR/). We recommend these tools because of their relatively deep validation based on the software engineering cores of the Insight Toolkit (ITK) http://www.itk.org/ and *R* http://cran.us.r-project.org/. Both of these resources, in which ANTs and ANTs*R* cores exist, are tested nightly on multiple platforms that involve both unit tests and tests for memory leaks using valgrind or related tools. Scientific software requires continual testing and maintenance to ensure the validity of scientific results that depend on correctness; regression testing is also crucial for maintaining reproducibility^41^.

An additional reason that we recommend dependence on scientific software with continual testing and a consistent implementation and definition of physical space is to enable data/modality fusion. As noted by Chris Gorgolewski in personal communication, ‘What is LPS in ITK world is RAI in NIPY or FreeSurfer world.’ These inconsistencies can lead to artificial findings when trying to combine processing methods across different analysis systems. Thus, we have sought to build a complete analysis pipeline that accommodates the PTBP modalities in an integrated manner.

This document is compilable markup that, when combined with the demographics files, produces this pdf via knitr http://yihui.name/knitr/. The source file for this document is stored within the Figshare repository for this article (Data Citation 1). Dependencies for both data acquisition and analyses are listed here. Software which we have permission to redistribute is within the Figshare site.

- http://cran.us.r-project.org/ - statistics.
- http://stnava.github.io/ANTsR/ - imaging specific I/O, statistical models and data curation with *R*.
- http://stnava.github.io/ANTs/ - template building, image registration and segmentation tools wrapped by ANTs*R* for *R*.
- http://www.itk.org/ - image processing library.
- http://www.itksnap.org/ - for visualization and semi-automated segmentation^113^.
- All analyses were performed on OSX or Linux operating systems.
- The associated code files are provided under the CC BY licence.

While we make every effort to achieve a reproducible analysis across all platforms, some operating systems are less amenable to this goal. Unix-alikes are most reliable for the software referenced here.

## Additional information

**How to cite this article:** Avants, B. B. *et al.* The pediatric template of brain perfusion. *Sci. Data* 2:150003 doi: 10.1038/sdata.2015.3 (2015).

## Supplementary Material



Supplementary Information

## Figures and Tables

**Table 1:** Age modeling from structural imaging variables.

	**Estimate**	**Std. Error**	***t*** **value**	**Pr(>|t|)**
(Intercept)	12.3390	4.6227	2.67	0.0091
BStem	0.0002	0.0001	3.01	0.0034
CBF	−0.0319	0.0118	−2.71	0.0081
FA	74.6610	10.9782	6.80	0.0000
Thickness	−6.0487	1.0827	−5.59	0.0000
DGM	−0.0001	0.0000	−3.62	0.0005
Cross-validation shows that this simple general linear model can predict age with accuracy of 1.61+/−0.15 years when training on half of the data and testing on the other half, as assessed by 100 data resamplings. Using multiple modalities to predict age shows that each modality contributes useful information even in the presence of other imaging measurements.				
