# Supplementary material for: The pediatric template of brain perfusion
Source: Sci Data. 2015 Feb 3;2:150003. doi: 10.1038/sdata.2015.3 (PMC4413243; doi:10.1038/sdata.2015.3)
Supplement: Supplementary Information [file sdata20153-s2.pdf]

Supplementary Figures

Supplemental figure 1. Page 2.
Supplemental figure 2. Page 3.
Supplemental figure 3. Page 4.
Supplemental figure 4. Page 5.

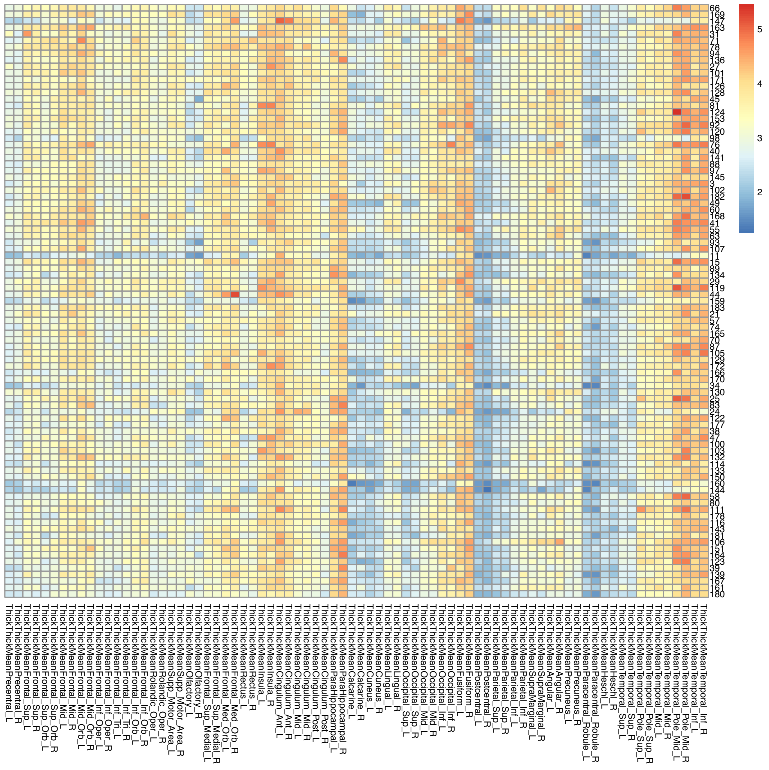

Supplemental Figure 1. Correlations among the PTBP thickness meausurements. The mean ROI-wise cortical thickness across all cross-sectional subjects with complete multiple modality data (n=91) based on the AAL labels. Subjects are sorted from youngest (first row, age 7) to oldest (last row, age 18).

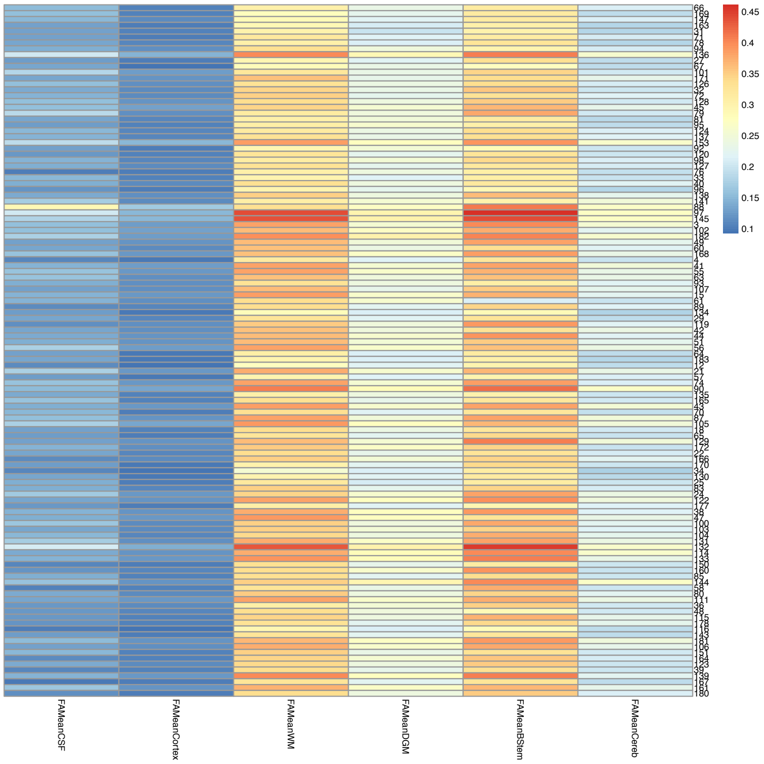

Supplemental Figure 2. Correlations among the PTBP FA meausurements. The mean ROI-wise fractional anisotropy across all subjects where the white matter definition is based on the 6-tissue segmentation. Subjects are sorted from youngest (first row, age 7) to oldest (last row, age 18)

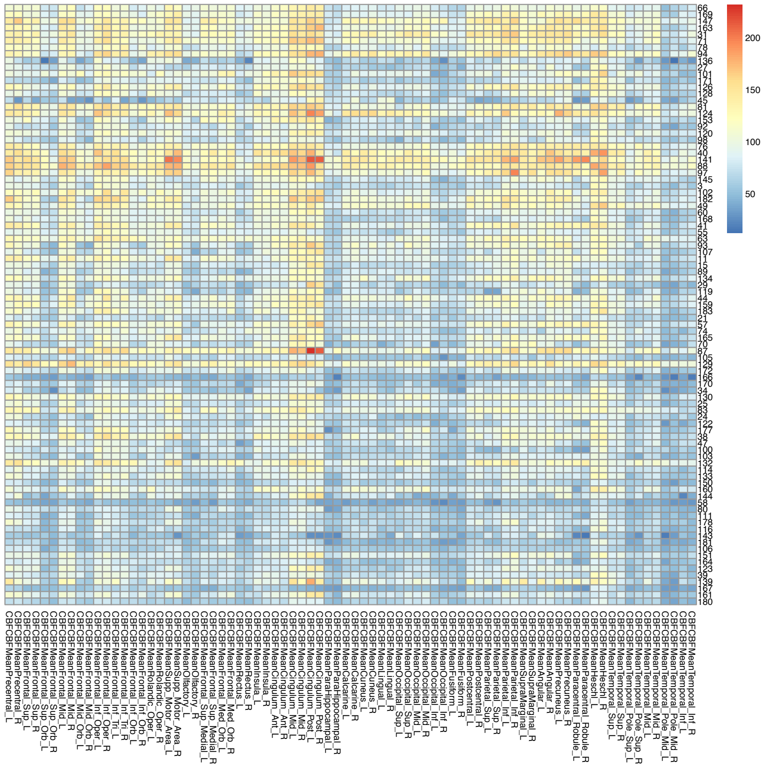

Supplemental Figure 3. Correlations among the PTBP CBF measurements. The mean ROI-wise CBF across all cross-sectional subjects with complete multiple modality data (n=91) based on the AAL labels. At right, the mean ROI-wise BOLD network connectivity in each AAL region based on subject-specific network analysis. Subjects are sorted from youngest (first row, age 7) to oldest (last row, age 18).

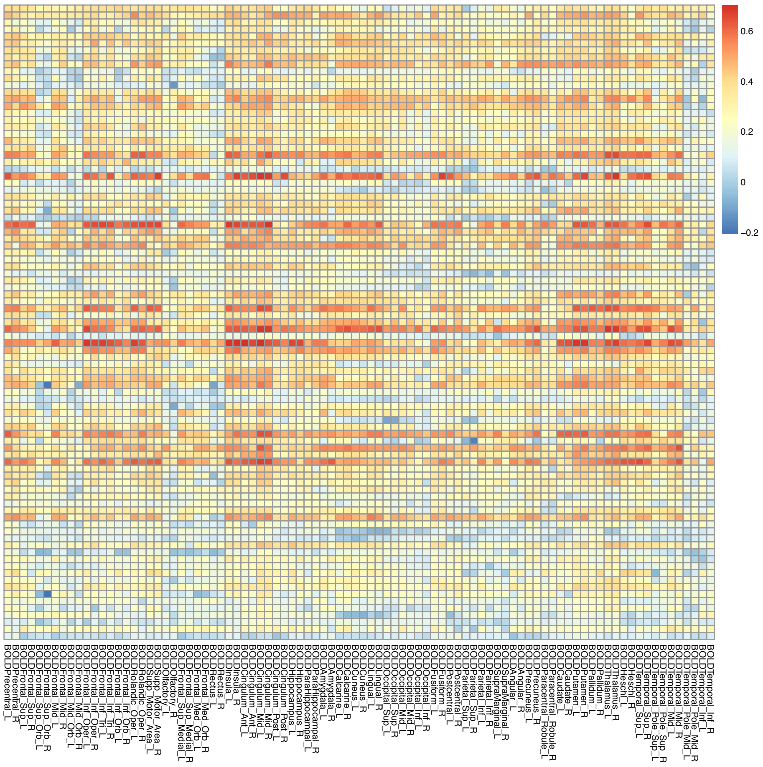

Supplemental Figure 4. Correlations among the PTBP BOLD connectivity measurements. The mean ROI-wise BOLD network connectivity in each AAL region is based on subject-specific network analysis. Subjects are sorted from youngest (first row, age 7) to oldest (last row, age 18).
